# The barley *DIR* gene family: An expanded gene family that is involved in stress responses

**DOI:** 10.3389/fgene.2022.1042772

**Published:** 2022-11-02

**Authors:** Ruihan Luo, Wenqiu Pan, Wenqiang Liu, Yuan Tian, Yan Zeng, Yihan Li, Zhimin Li, Licao Cui

**Affiliations:** ^1^ College of Bioscience and Engineering, Jiangxi Agricultural University, Nanchang, Jiangxi, China; ^2^ State Key Laboratory of Crop Stress Biology in Arid Areas and College of Agronomy, Northwest A&F University, Yangling, Shaanxi, China; ^3^ Xintai Urban and Rural Development Group Co., Ltd., Taian, Shandong, China

**Keywords:** barley, gene family expansion, dirigent gene family, domestication, expression patterns

## Abstract

Gene family expansion plays a central role in adaptive divergence and, ultimately, speciation is influenced by phenotypic diversity in different environments. Barley (*Hordeum vulgare*) is the fourth most important cereal crop in the world and is used for brewing purposes, animal feed, and human food. Systematic characterization of expanded gene families is instrumental in the research of the evolutionary history of barley and understanding of the molecular function of their gene products. A total of 31,750 conserved orthologous groups (OGs) were identified using eight genomes/subgenomes, of which 1,113 and 6,739 were rapidly expanded and contracted OGs in barley, respectively. Five expanded OGs containing 20 barley dirigent genes (*HvDIR*s) were identified. *HvDIR*s from the same OG were phylogenetically clustered with similar gene structure and domain organization. In particular, 7 and 5 *HvDIR*s from OG0000960 and OG0001516, respectively, contributed greatly to the expansion of the DIR-c subfamily. Tandem duplication was the driving force for the expansion of the barley *DIR* gene family. Nucleotide diversity and haplotype network analysis revealed that the expanded *HvDIR*s experienced severe bottleneck events during barley domestication, and can thus be considered as potential domestication-related candidate genes. The expression profile and co-expression network analysis revealed the critical roles of the expanded *HvDIR*s in various biological processes, especially in stress responses. *HvDIR18*, *HvDIR19*, and *HvDIR63* could serve as excellent candidates for further functional genomics studies to improve the production of barley products. Our study revealed that the *HvDIR* family was significantly expanded in barley and might be involved in different developmental processes and stress responses. Thus, besides providing a framework for future functional genomics and metabolomics studies, this study also identified *HvDIR*s as candidates for use in improving barley crop resistance to biotic and abiotic stresses.

## Introduction

Based on sequence similarity, a large repertoire of protein-coding genes can be categorized into gene families (Vakirlis et al., 2020). Family members are defined as genes that share a coded protein domain (Jamsheer et al., 2015). For example, the *bZIP* family genes all contain a highly conserved bZIP domain composed of a basic DNA binding region and an adjacent leucine zipper, and these are important regulators of biological processes in plants, such as growth and development and stress-induced responses (Nijhawan et al., 2008). Generally, these proteins function by forming stable three-dimensional structures through their conserved domains.

Families of genes originate from a common ancestor gene that has undergone iterations of sequence duplication and mutation events (Li et al., 2022). Gene members within the same family can be closely localized at a given interval to form gene clusters. However, in most cases, gene members have been found to be enriched in locations that lie far away from one another, on either the same or different chromosomes (Wang et al., 2018). Gene duplication and sequence loss are considered the primary factors driving evolutionary novelty, as the original genetic material provides the basis upon which evolution can progress through natural selection (Ohta, 2003). Duplicated genes occur primarily through two prominent mechanisms: small-scale duplications (SSDs), and whole-genome duplications (WGDs) (Hanada et al., 2018). Most WGDs have been found to occur during the period surrounding the Cretaceous-Paleogene extinction event (Vanneste et al., 2014). SSD events encompass tandem, segmental, and transposon-mediated duplications, and these have arisen continuously and at high frequencies during the course of plant evolution (Rostoks et al., 2005). The expansion and contraction of various gene families have altered their sizes across lineages and functions to drive natural variation for phenotypic diversity and plant speciation across the plant kingdom. The evolutionary success of seed and flowering plants has universally been related to gene duplication events (Tautz and Domazet-Lošo, 2011; Hanada et al., 2018). For example, the expansion of the chalcone synthase (*CHS*) family is involved in the biosynthesis of urushiols and their related phenols (Wang et al., 2020). Large-scale gene family expansion in elephant grass has been associated with its rapid growth, drought tolerance, and biomass accumulation (Zhang et al., 2022).

The term “dirigent” (DIR) is derived from the Latin term “dirigere” (to align or guide), and dirigent proteins function to guide the proper biochemical construction of compounds. DIR proteins were first isolated from *Forsythia intermedia* (Gang et al., 1999). They are highly involved in the biosynthesis of lignin oligomers through directing the free radical coupling of E-coniferyl alcohol, whereby monomers are linked by the C8 central carbon atoms of propyl side chains (Gang et al., 1999; Burlat et al., 2001; Dalisay et al., 2015). Members of *DIR* genes have been identified and characterized in various plant species; 26 have been identified in *Arabidopsis thaliana* (*A. thaliana*) (Liao et al., 2017), 54 in rice (*Oryza sativa*) (Liao et al., 2017; Song and Peng, 2019), 35 in sitak spruce (*Picea sitchensis*) (Ralph et al., 2007), 24 in pepper (*Capsicum annuum*) (Khan et al., 2018), 45 in barrel medic (*Medicago truncatula*) (Song and Peng, 2019), and 54 in soybean (*Glycine max*) (Ma et al., 2021). Initially, DIR proteins were divided into five subfamilies: DIR-a, DIR-b, DIR-c, DIR-d, and DIR-e (Ralph et al., 2007). Following an increase in the known members of DIR proteins, the DIR-b and DIR-d subfamilies were subsequently grouped together, while the DIR-f and DIR-g subfamilies have since come into existence (Ralph et al., 2006). Based on phylogenetic relationships, the DIR-c subfamily is known to be specific to monocots (Corbin et al., 2018). Proteins from the DIR-a subfamily are reported to be involved in the formation of pinoresinol. Outside of these two subfamilies, the biochemical and/or physiological functions of other subfamily members remain to be elucidated (Corbin et al., 2018).

The involvement of DIR proteins in lignin biosynthesis is known to positively contribute to plant defense (Gallego-Giraldo et al., 2011). Increasing evidence suggests that DIR proteins play essential roles in stress-induced responses, especially in response to plant pathogens. For example, the overexpression of *OsJAC1* promotes plant resistance against a broad range of pathogenic infections (Weidenbach et al., 2016). The overexpression of *GHDIR1* in cotton was found to confer enhanced resistance to the spread of *Verticillium dahlia* (Shi et al., 2012). The expression of *DIR* genes alters the response of canola plants against broad-spectrum fungal pathogens, including *Leptosphaeria maculans* and *Rhizoctonia solani* (Wang and Fristensky, 2001). *TaDIR13* increases the biosynthesis of lignan and enhances resistance to pathogens in wheat (*Triticum aestivum*) (Ma and Liu, 2015). Beyond pathogen responses, DIR proteins are also involved in plant growth, development, and responses to abiotic stresses. In *Boea hygrometrica*, *BhDIR1* expression was found to be induced by multiple abiotic stresses, such as CaCl_2_, H_2_O_2_, abscisic acid (ABA), and dehydration (Wu et al., 2009). The silencing of *CaDIR7* in pepper was found to weaken plant defense to both NaCl and mannitol-induced stress (Khan et al., 2018). *A. thaliana* ESB1, a dirigent-domain-containing protein, plays an essential role in ensuring the correct formation of lignin-based Casparian strips in the root (Hosmani et al., 2013). In soybean, *Pdh1* controls pod dehiscence by increasing the torsion of pod walls under low humidity. The overexpression of *GMDIR27* increased pod dehiscence by regulating the expression of pod dehiscence-related genes (Ma et al., 2021).

As one of the first domesticated crops originating from the Fertile Crescent approximately 10,000 years ago, barley (*Hordeum vulgare*) today ranks the fourth cereal crop in terms of both its production and growing hectares (Ma et al., 2021). The vast majority of barley production is used to produce animal feed and both alcoholic and non-alcoholic beverages. Approximately 5% is devoted to human consumption as an ingredient in a wide range of food products (Ullrich, 2010). Barley is a staple food resource in remote areas characterized by severe environments because of its hardiness and strong adaptive plasticity (Grando and Macpherson, 2005). Owing to its unique health-promoting benefits, the particular type of dietary fiber derived from barley has attracted attention from researchers in recent years (Bader Ul Ain et al., 2019).

Clustering analysis found 31,750 orthologous groups (OGs) among 8 genomes/sub-genome, and 1,113 were found to be significantly expanded in barley. It should be noted that five OGs consisting of 20 barley *DIR* genes (*HvDIR*s) have undergone gene family expansion. *HvDIR*s within the same OGs show similar gene structure, domain organization, and closer relatedness than those among different OGs. An analysis of gene duplication revealed that tandem repeat events represent the driving force that has contributed to the expansion of *HvDIR*s. The expanded *HvDIR*s then suffered a severe genetic bottleneck during barley domestication, from which domesticated-related genes were then propagated. Our comprehensive analysis could serve as valuable information for characterizing the evolutionary trajectory of barley, and also contribute to subsequent functional studies on expanded genes within the barley genome.

## Materials and methods

### Gene family expansion and contraction

The protein sequences from six grass species were downloaded from their respective links: http://doi.org/10.5447/ipk/2021/3 (barley), http://plants.ensembl.org/index.html (*Aegilops tauschii*, *Brachypodium distachyon*, *Secale cereale*, and wheat), and http://www.mbkbase.org/Tu/(*Triticum urartu*). The hexaploid wheat was split into the A, B, and D subgenomes. An inhouse python script was written and run to obtain the longest transcript for each gene. OG clustering was performed by the OrthoFinder v2.5.4 program, using the “-m MSA” option (Emms and Kelly, 2019). A phylogenetic tree was constructed using a total of 7,180 single-copy genes in OrthoFinder. A calibrated species tree was generated using the r8s (http://loco.biosci.arizona.edu/r8s/) software with the TN algorithm and the penalized likelihood method. The TimeTree (http://www.time.org/) database was used to obtain the divergence time calibration information between barley and *B. distachyon* (median time = 32 MYA). Highly variable OGs were excluded from subsequent analysis. Gene family expansion and contraction analysis were performed using the CAFÉ v4.2 software (De Bie et al., 2006). Gene gain or loss along each lineage across the species-specific phylogenetic tree was identified under a random birth and death model using the maximum likelihood method.

### Identification of *DIR* genes in barley

The hidden Markov model (HMM) of the DIR domain (PF03018) was downloaded from the Pfam database. The HMM profile was used to search against the barley genomic proteins using the HMMER v3.3.2 software with default inclusion threshold (0.001) (Yoon, 2009). The putative *DIR* genes were submitted to online HMMER (https://www.ebi.ac.uk/Tools/hmmer/), National Center for Biotechnology Information - Conserved Domains Database (NCBI-CDD: https://www.ncbi.nlm.nih.gov/cdd/), and Simple Modular Architecture Research Tool (SMART: http://smart.embl-heidelberg.de/) databases for domain validation. Candidate proteins without the DIR domains were excluded. The physicochemical properties, such as molecular weight (MW), protein length, theoretical isoelectric point (pI), and grand average of hydropathicity (GRAVY), were estimated using the online ExPASy tool (https://web.expasy.org/protparam/). Plant-Ploc v2.0 (http://www.csbio.sjtu.edu.cn/bioinf/plant/) was used to predict subcellular localization of *DIR* genes.

### Phylogenetic relationships and expansion pattern analysis

The chromosome location of barley *DIR* genes were visualized using the MapGene2Chrom (MG2C) software (http://mg2c.iask.in/mg2c_v2.0/). The validated *DIR* genes were designated as *HvDIR1* to *HvDIR64* according to their physical location and chromosome number. The Clustal X program was used to perform multiple sequence alignment using the full-length proteins from *A. thaliana*, rice, and barley. The neighbor-joining phylogenetic tree was generated by MEGA 11 with 1,000 bootstrap computations. The exon-intron organization of *HvDIR*s are displayed using the Gene Structure Display Server v2.0 (http://gsds.cbi.pku.edu.cn/) based on the gene transfer format file. An all vs. all Local Alignment Search Tool (BLAST) was used to determine the expansion patterns of *HvDIRs* using the following criteria: 1) the identity of the aligned region should be no less than 70%; 2) the shorter sequence should cover more than 70% of the longer sequence (Gu et al., 2002). In addition, duplicated genes are linked by colored lines using Circos v0.69-8 (Krzywinski et al., 2009). The codeml program of PAML v4.3 was used to evaluate the nonsynonymous (Ka), synonymous (Ks) substitution rates, and the Ka/Ks ratios were calculated using the codeml subroutine in PAML v4.3 (Yang, 2007). The divergence time of the duplicated gene pairs was estimated using formula T = Ks/2λ (where *λ* = 6.5 × 10^–9^).

### Nucleotide variants and genetic diversity of *HvDIR*s

The barley exome-captured resequencing data were retrieved from the NCBI Sequence Read Archive (SRA) database (https://www.ncbi.nlm.nih.gov/). The clean reads were aligned to the barley reference genome using the BWA v0.7.17 software. The Picard v2.27.4 tool (http://broadinstitute.github.io/picard) was used to sort mapped reads and mark PCR duplicates. Single nucleotide polymorphism (SNP) calling was performed using the GATK software in default mode. Nucleotide variants were annotated by SnpEff v5.1 (https://pcingola.github.io/SnpEff/). The following criteria were used for SNP filtration: 1) minor allele frequency (MAF) > 0.05; 2) a maximum missing rate should be lower than 0.2 for a single SNP locus and for individual accession. The final dataset consisted of 128 landraces and 77 wild barleys (Supplementary Table S1). To better elucidate the evolutionary history of *HvDIR*s, variants within the exon were retained for subsequent analysis. The phylogenetic tree was constructed using Treebest v1.9.2. Population structure was inferred using ADMIXTURE (http://www.genetics.ucla.edu/software/admixture/) with K ranging from 2 to 5. The smartpca subroutine in EIGENSOFT v4.2 was used to perform the principal component analysis (PCA). The nucleotide diversity (π) was estimated using vcftools v0.1.16. Haplotype statistics were performed for each *HvDIR* using DNAsp v6.12.01 software. The transmission network was generated using the PopART v1.7 package with the media-joining method.

### *Cis*-acting element identification and expression profile analysis of *HvDIR*s

The 1.5 kb genomic sequences upstream of the coding regions were extracted and screened by the PlantCARE tool (http://bioinformatics.psb.ugent.be/webtools/plantcare/html/). Potential microRNA (miRNA) binding sites were predicted by the psRNATarget online server (http://plantgrn.noble.org/psRNATarget/). A total of four publicly available RNA-sequencing (RNA-seq) bio-projects composed of 166 RNA-seq libraries were downloaded from the NCBI SRA database. RNA-seq of tissues from 16 developmental stages, including developing grain (15/5 days after pollination), embryonic tissue (4 days), epidermal strips (4 weeks after pollination), etiolated seedling (10 days old after planting), 5-mm developing inflorescences, 1-cm developing inflorescences, 10-cm shoots from seedlings, inflorescences, lemma (6 weeks after pollination), lodicule (6 weeks after pollination), developing tillers at third stem internode (6 weeks after pollination), dissected inflorescences, palea (6 weeks after pollination), rachis (5 weeks after pollination), roots from 17- and 28-day old seedlings (after planting), roots (4 weeks after pollination), and senescing leaves (8 weeks after pollination), was used to determine the spatiotemporal expression patterns of the expanded *HvDIR*s (Bio-project: PRJEB14349) (Supplementary Table S2) (Mascher et al., 2017). For drought with heat treatment, two barley genotypes, a modern cultivar (Scarlett) and an outstanding Spanish landrace-derived inbred line (SBCC073), were subjected to greenhouse drought with an intermediate drought stress level of 50% field capacity and max temperature >28°C for 20 days (Bio-project: PRJEB12540) (Cantalapiedra et al., 2017). For salt treatment, barley plants were salt-treated by adding 50 mM NaCl per day to achieve a final concentration of 100 mM in hydroponics. The shoots and roots of plant samples collected from each treatment were separated after 9 days of salt treatment (Bio-project: PRJNA546269) (Fu et al., 2019). For low nitrogen treatment, the seedlings of two barley genotypes BI-04 and BI-45 were treated with low nitrogen nutrient solution (0.24 mM ammonium nitrate) when the fourth leaf appeared, and new shoots were sampled immediately (0 h) or 1h and 24 h after the beginning of the low nitrogen treatment (Bio-project: PRJNA400519) (Chen et al., 2018). For aluminum and low pH stress treatment, the root meristem of barley seedlings were grown under the optimal pH (6.0) and low pH (4.0) and aluminum (10 mM bioavailable Al^3+^ions) water culture conditions in two independent, short-term (24 h) and long-term (7 days), experiments (Bio-project: PRJNA704034) (Szurman-Zubrzycka et al., 2021). Clean reads were mapped to the barley reference genome using the Hisat2 v2.2.1 software (Pertea et al., 2015), and the corresponding gene expression levels, measured as the fragments per kilobase of transcript per million fragments mapped reads (FPKM) value, were evaluated by StringTie v1.3.5 (Pertea et al., 2015). FPKM values with log_2_ (FPKM+1) normalization were visualized using the pheatmap package in R.

### Co-expression network construction and enrichment analysis

In order to determine the genes co-expressed with *HvDIR*s, three additional bio-projects (PRJNA324116, PRJNA744693, and PRJNA439267) consisting of 82 RNA-seq libraries were added to the current transcriptome datasets (Pacak et al., 2016; Kreszies et al., 2019; Chen et al., 2021). The Weighted Gene Co-Expression Network Analysis (WGCNA) R package was used for co-expression network analysis. The co-expressed genes were annotated using the KOBAS v3.0 database (http://kobas.cbi.pku.edu.cn/kobas3/), and the unannotated genes were not represented in the network. The top 30 co-related genes were considered candidates. Cytoscape v3.8.0 platform was used to visualize the co-expression networks. Gene ontology (GO) and Kyoto Encyclopedia of Genes and Genomes (KEGG) databases were used to perform functional annotation using EGGNOG-MAPPER v2 (http://eggnog-mapper.embl.de/) with default parameters (Cantalapiedra et al., 2021). The GO term and KEGG pathway enrichment analyses were performed using Tbtools v1.098726 (Chen et al., 2020). The GO terms and KEGG pathways with Qvalue ≤0.05 were considered as significantly enriched. The top-ranking words of GO and KEGG were visualized using the ggplot2 package in R.

## Results

### OG clustering, phylogenomic analysis, and gene family expansion and contraction analysis

**FIGURE 1:**
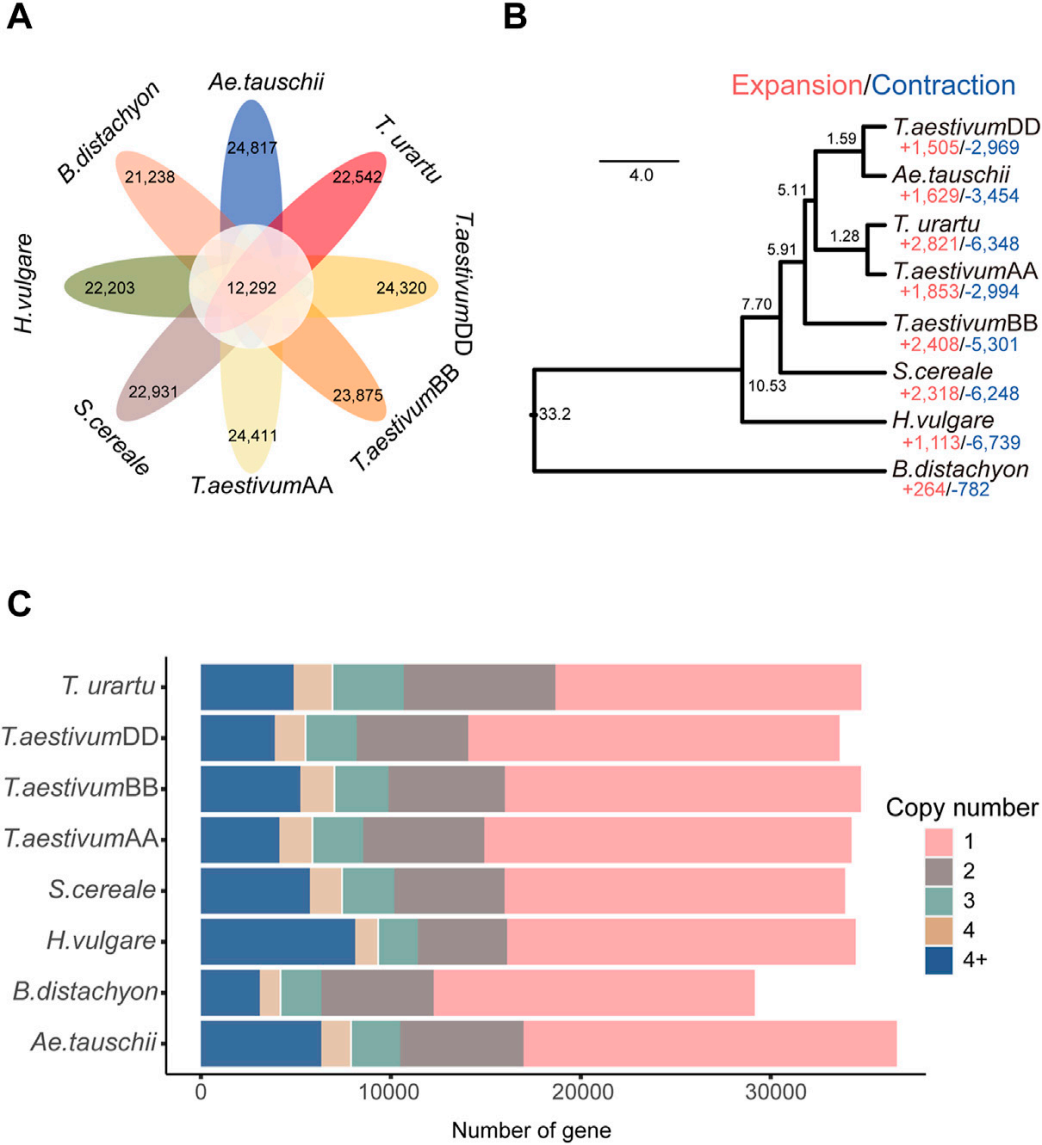
Gene clustering and phylogenetic analysis. **(A)** Petal diagram of the OGs of 8 genomes/subgenomes; **(B)** CAFÉ-based estimates of gene family expansions and contractions. The numbers after “+” and “−” represent the number of expanded and contracted gene families, respectively; **(C)** Copy-number distribution of the OGs.

OG clustering was performed using the genomic sequences from the genomes/subgenomes of eight grass species (*T. urartu*, wheat AA, wheat BB, *Ae. tauschii*, wheat DD, rye, barley, and *B. distachyon*). A total of 31,750 OGs were identified. Among them, 12,292 OGs were shared among all the genomes/subgenomes (Supplementary Table S3, Figure 1A). Copy number analysis revealed that the OGs in barley were present at various ranges of copy number, from one to more than four copies, and genes present as single-copy genes in OGs represented the largest proportion of genes (Figure 1C). Based on 7,180 single-copy genes, the phylogenomic analysis showed that the outgroup taxa of *B. distachyon* was phylogenetically distant from the other genomes/subgenomes. The wheat AA and wheat BB subgenomes were mapped as the sister branches to their ancestor species *Ae. tauschii* and *T. urartu*, respectively. The phylogenetic position of barley was found to be located between rye and *B. distachyon*, which is consistent with the topology of the taxonomic tree reported in previous studies (Li et al., 2021a).

In total, 22,203 out of 31,750 OGs were found in barley. Among them, there were 1,113 expanded OGs and 6,739 contracted OGs (*p* ≤ 0.05) (Figure 1B). The GO term enrichment analysis showed that the expanded-OG related genes were enriched in peptidyl-threonine phosphorylation (GO:0018107), defense response (GO:0006952), response to UV-A (GO:0070141) and positive regulation of seed germination (GO:0010030) in the biological process terms; DNA insertion or deletion binding (GO:0032135), electron transfer activity (GO:0009055), and NADPH dehydrogenase activity (GO:0003959) in the molecular function terms; photosynthetic membrane (GO:0034357), thylakoid membrane (GO:0042651), and plastid thylakoid (GO:0031976) in the cellular component terms (Supplementary Table S4; Supplementary Figure S1A). The KEGG pathway enrichment analysis showed that homologous recombination (KO03440), photosynthesis proteins (KO00194), mitochondrial biogenesis (KO03029), and oxidative phosphorylation (KO00190) pathways were significantly enriched (Supplementary Table S5; Supplementary Figure S1B).

### Genome-wide identification of the expanded *DIR* gene family

The HMMER search against the barley proteins identified 65 putative *DIR* genes. The online SMART, NCBI-CDD, and HMMER databases verified that 64 of these *DIR* genes had DIR domains (Supplementary Table S6). The validated genes were named as *HvDIR1* to *HvDIR64* according to their physical location from chromosomes 1 to 7 (Supplementary Table S7). Proteins encoded by *HvDIRs* ranged in amino acid length from 162 (*HvDIR25*) to 392 (*HvDIR41*), with the MW ranging from 16.81 (*HvDIR25*) to 42.21 (*HvDIR41*) kDa and the pI ranging from 4.70 (*HvDIR31*) to 11.19 (*HvDIR48*). The GRAVY ranged from -0.47 (*HvDIR14*) to 0.41 (*HvDIR44*). The GRAVY of 29 DIR proteins were negative, which demonstrated their hydrophilic properties.

Among the expanded OGs, a total of five OGs composed of 20 *HvDIR*s were found to be significantly expanded in barley, namely OG0000960 (7 *HvDIR*s, expansion number 4), OG0001516 (5 *HvDIR*s, expansion number 2), OG0002286 (3 *HvDIR*s, expansion number 1), OG0002727 (3 *HvDIR*s, expansion number 1) and OG0017605 (2 *HvDIR*s, expansion number 1) (Supplementary Table S8). Remarkably, while seven *HvDIR*s were identified in OG0000960, only 3 and 4 *DIR*s were found in *Ae. tauschii* and rye, respectively. In OG0001516, five *DIR*s each were identified in the wheat DD and barley genome/subgenome. A total of three *DIR*s were identified in OG0002286. The *DIR*s in OG0002727 were specific to the barley, and wheat AA subgenomes with 3 *DIR*s per OG per genome/subgenome. In comparison, only two *HvDIR*s were identified in OG0017605 (Supplementary Table S9).

### Phylogenetic relationships of the expanded *DIR*s in barley

**FIGURE 2:**
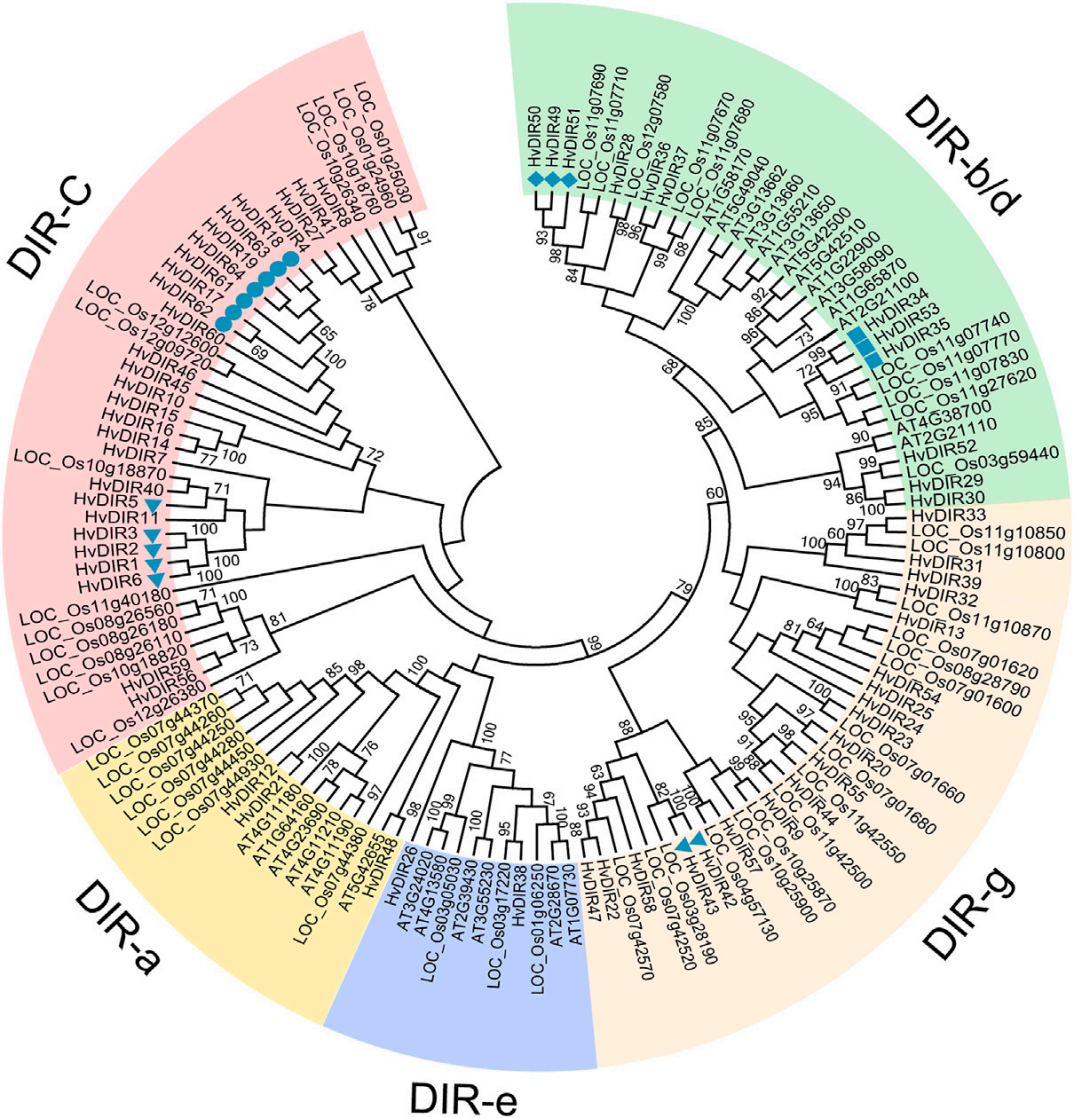
Phylogenetic analysis of DIR proteins from *A. thaliana*, rice, and barley. The phylogenetic tree was constructed using the NJ method with 1,000 bootstrap replications. The five subfamilies are indicated with different colors.

In order to determine the evolutionary relationships of the expanded *HvDIR*s, phylogenetic analysis of the *HvDIRs* was performed by comparing them with those of *A. thaliana* and rice (Figure 2). Based on their sequence similarity, the 139 *DIR*s were classified into 5 subfamilies. It should be noted that DIR-c, a specific subfamily for monocots, had no *DIR*s from *A. thaliana* (Corbin et al., 2018). *HvDIR*s within the same OG were tightly grouped into the same cluster, as follows: OG0000960 (*HvDIR17*, *HvDIR18*, *HvDIR19*, *HvDIR61*, *HvDIR62*, *HvDIR63*, and *HvDIR64*) and OG0001516 (*HvDIR1*, *HvDIR2*, *HvDIR3*, *HvDIR5*, and *HvDIR6*) were assigned into the DIR-c subfamily; OG0002727 (*HvDIR34*, *HvDIR35*, and *HvDIR53*) and OG0002286 (*HvDIR49*, *HvDIR50*, and *HvDIR51*) were grouped into the DIR-b/d subfamily; *HvDIR42* and *HvDIR43* of OG0017605 were assigned to the DIR-g subfamily.

**FIGURE 3:**
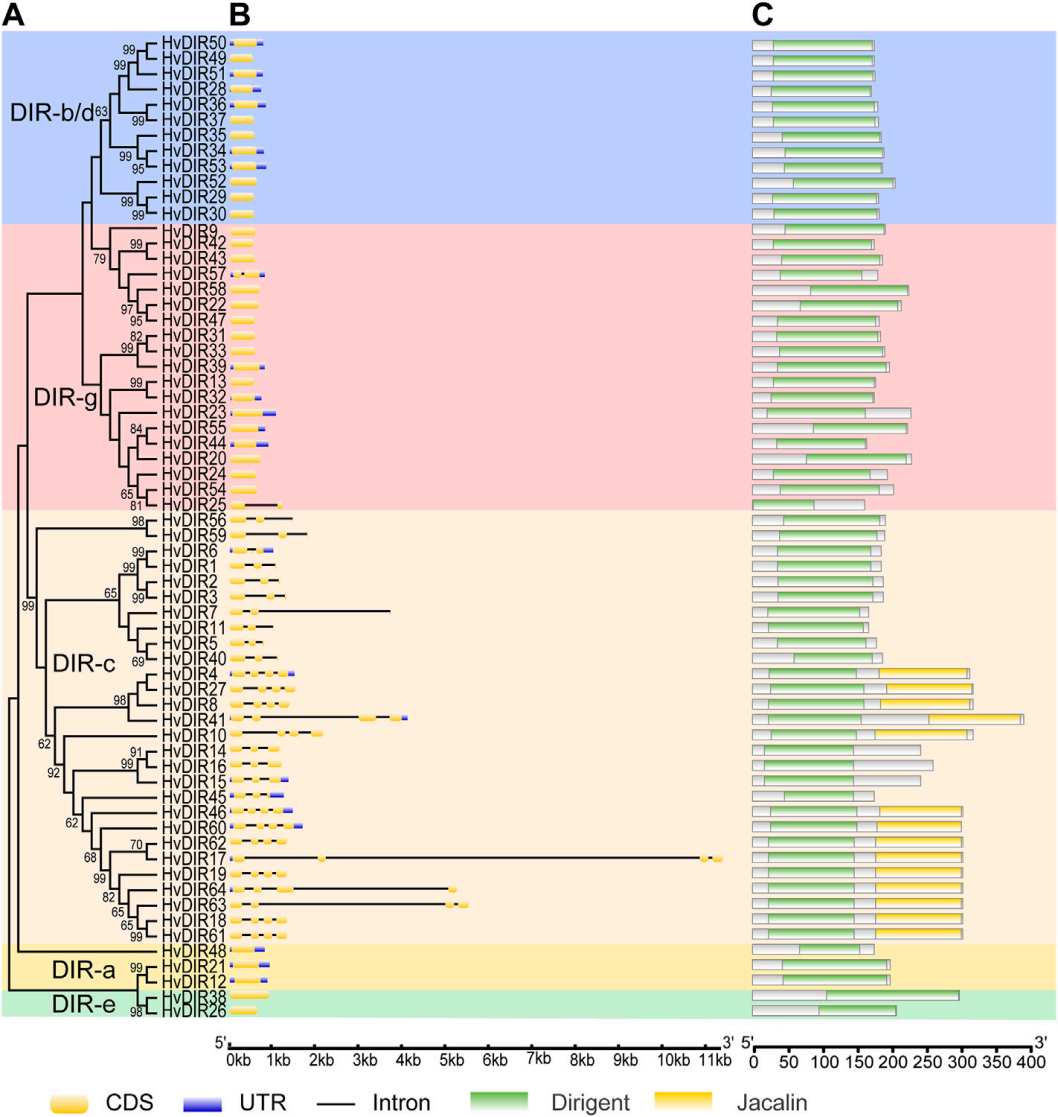
Phylogenetic tree, exon-intron structure, and conserved domains of *HvDIR*s. **(A)** Phylogenetic tree for each subfamily; **(B)** Gene structure of *HvDIR*s. CDSs are indicated in faint yellow. Black lines represent introns; **(C)** The conserved domains of HvDIRs. Dirigent domain is indicated in green. Jacalin domain is indicated in yellow.

To gain a greater insight into the evolutionary relationships of the expanded *HvDIR*s, the exon-intron structures and conserved domains were analyzed (Figure 3). Genes within the same OGs tended to have similar gene structure and domain organization, whereas they varied greatly between different OGs. For example, three *HvDIR*s of OG0002286 and two *HvDIR*s of OG0017605 were found to be intron-less and their exons were similar in length, whereas genes within OG0000960 had four exons and all of them had an additional jacalin domain.

### Chromosomal location and expansion patterns of *HvDIR*s

Chromosomal location showed that *HvDIR*s were unevenly distributed across the seven chromosomes of barley, and chromosome 2H contained the highest number of *HvDIR*s (16 genes), followed by chromosome 5H (12 genes) and 4H (11 genes) (Supplementary Figure S2). The expanded OG-associated *HvDIR*s were mainly found at the distal end of the chromosome arms, including chromosome 1H, 2H, 4H, and 5H. It should be noted that *HvDIR61*, *HvDIR62*, *HvDIR63*, and *HvDIR64* were located on the unanchored super-scaffolds (chrUn).

**FIGURE 4:**
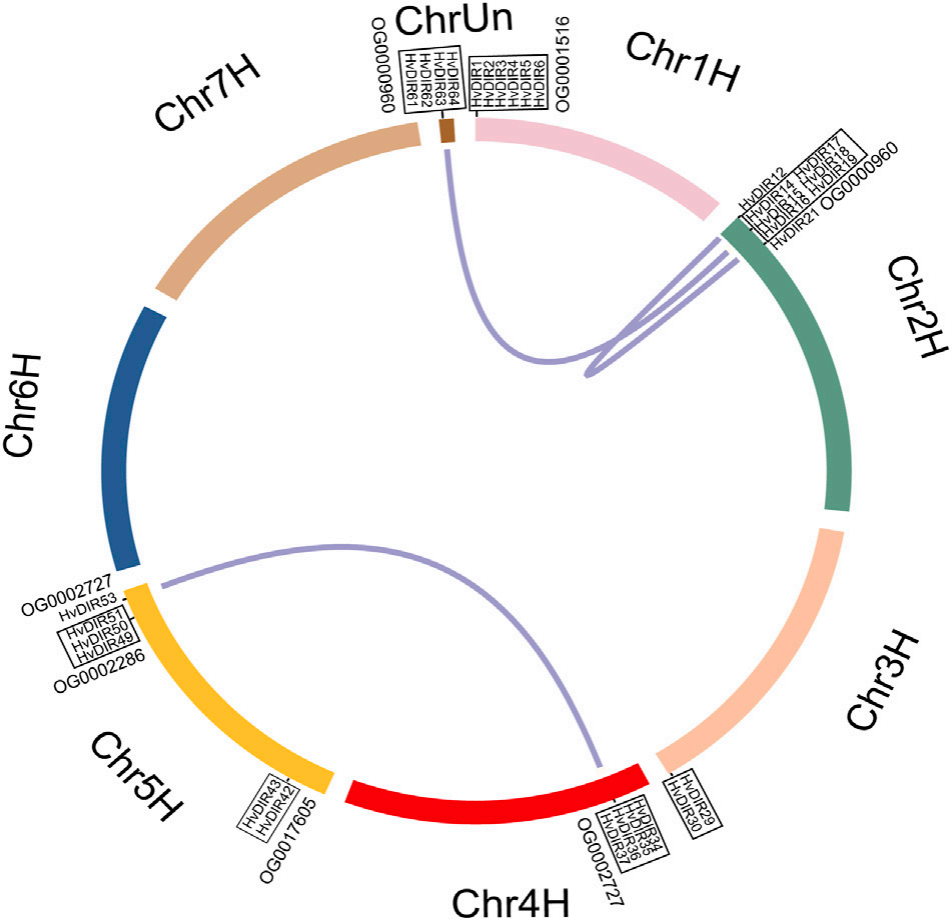
Chromosomal location and gene duplication of *HvDIR*s. Tandemly duplicated gene pairs are highlighted with black boxes.

Gene duplications were further found to show the expansion pattern of *HvDIR*s. In total, three segmental duplication pairs and seven tandem duplication blocks were detected (Figure 4). On chromosome 1H, six *HvDIRs* (*HvDIR1*, *HvDIR*2, *HvDIR3*, *HvDIR4*, *HvDIR5*, and *HvDIR6*) formed a tandem duplication block. Except for *HvDIR4*, the remaining genes belonged to OG0001516. Genes within OG0000960 formed two tandem blocks on chromosomes 2H and Un. Notably, genes on chromosome Un were not located on definite chromosomes, further research is required to clarify their definite locations. *HvDIRs* in OG0002727 were related to a tandem duplication event on chromosome 4H, and a segmental event between 4H and 5H. Furthermore, two tandemly repeated blocks, including *HvDIR42* and *HvDIR43* from OG0017605, and *HvDIR49*, *HvDIR50*, and *HvDIR51* from OG0002286, were found to be present on the long and short arm of chromosome 5H, respectively. A total of 34 duplicated pairs were calculated for the expansion-related *HvDIR*s. The calculation of the Ka/Ks ratios to elucidate the evolutionary pressure of the duplicated gene pairs revealed that the Ka/Ks ratios of the duplicated pairs ranged from 0.06 to 0.59, suggesting that these genes experienced purifying selection during the expansion process (Supplementary Table S10).

### Population structure, genetic diversity, and haplotype network of the expanded *HvDIR*s

**FIGURE 5:**
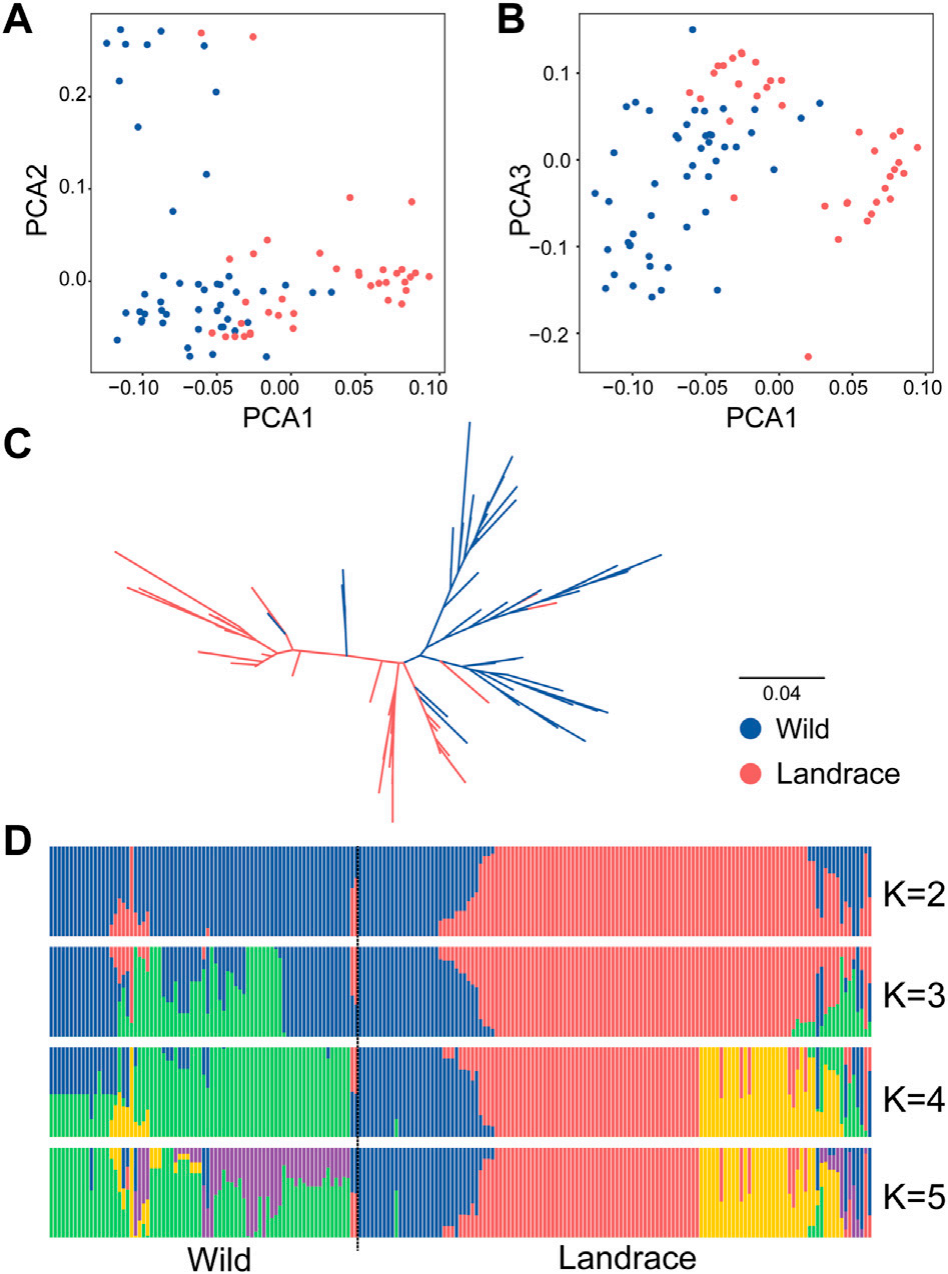
Population structure of wild barleys and landraces based on *HvDIR*-related SNPs. **(A)** Principal component analysis PC1 vs. PC2; **(B)** Principal component analysis PC1 vs. PC3; **(C)** The NJ phylogenetic tree; **(D)** Population structure with K ranging from 2 to 5.

**FIGURE 6:**
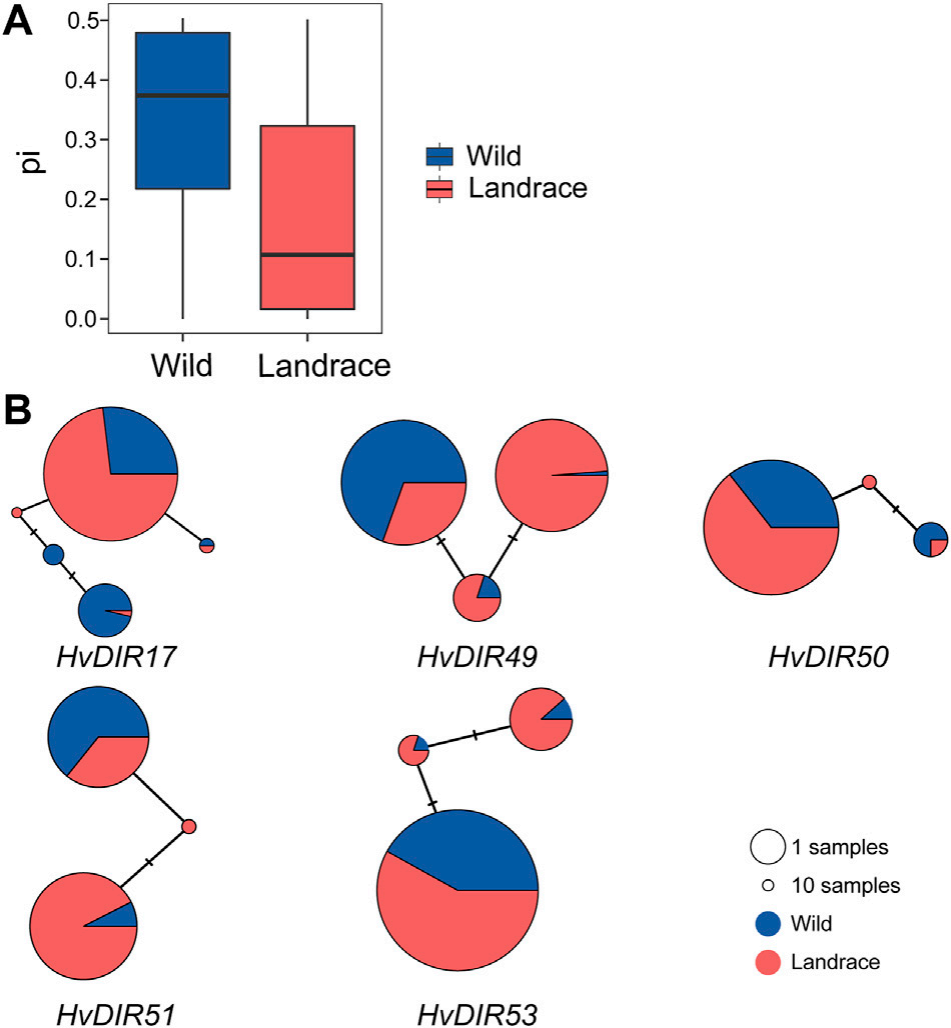
Genetic diversity and haplotype analysis of *HvDIRs*. **(A)** Genetic diversity; **(B)** Haplotype network of the expanded *HvDIRs*.

The variation landscape of the expanded *HvDIR*s was profiled using the exome-captured resequencing data of barley. We identified a total of 1,346 expanded *HvDIR*-related SNPs, including 446 missense variant, 363 synonymous variant, 273 intron variant, 190 3′-prime UTR variant, 66 5′-prime UTR variant, 5 stop gained, 2 start lost, and 1 initiator codon variant (Supplementary Tables S11, S12). Further elucidation of the evolutionary history of the expanded *HvDIR*s during the process of barley domestication using ADMIXTURE analysis showed a clear separation between wild and landrace barley with K = 2 (Figure 5D). Increasing K to 5 provided additional insights. Within the landrace population, accessions were grouped into three distinct groups, and PCA corroborated these findings. The first eigenvector elucidated 76.31% of the total variance and mainly captured the differentiation between wild barley and landrace barley. The second and third eigenvectors elucidated 33.12% and 25.44% of the variance, respectively, and distinguished the accessions according to their geographical origins (Figures 5A,B and Supplementary Table S13). Furthermore, a neighbor joining (NJ) phylogenetic tree showed the same population affinity (Figure 5C). Based on the population classification, nucleotide diversity was assessed to evaluate the genetic bottleneck of *HvDIR*s during barley domestication. The genetic diversity of the expanded *HvDIR*s decreased by 45.20% changing from wild barley (0.3281) to landrace populations (0.1798) (Figure 6A).

According to the nucleotide variation, a total of 17 haplotypes of five expanded *HvDIR*s were obtained (Figure 6B). We observed significant genetic differentiation of haplotypes between wild barley and landraces. For *HvDIR17*, wild barleys had the TTC haplotype, while the CAA haplotype was specific to landraces. *HvDIR51* in wild barley had the dominant haplotype CC, whereas the proportion of wild barleys were greater than that of landraces for haplotype TT. Additionally, our results also revealed certain rare haplotypes in wild barley. The appearance of these nucleotide variations would help increase the haplotype polymorphism of wild barley. The haplotype divergence between wild barley and landrace reflects the differences due to artificial selection.

### Expression patterns of the expanded *HvDIR*s

**FIGURE 7:**
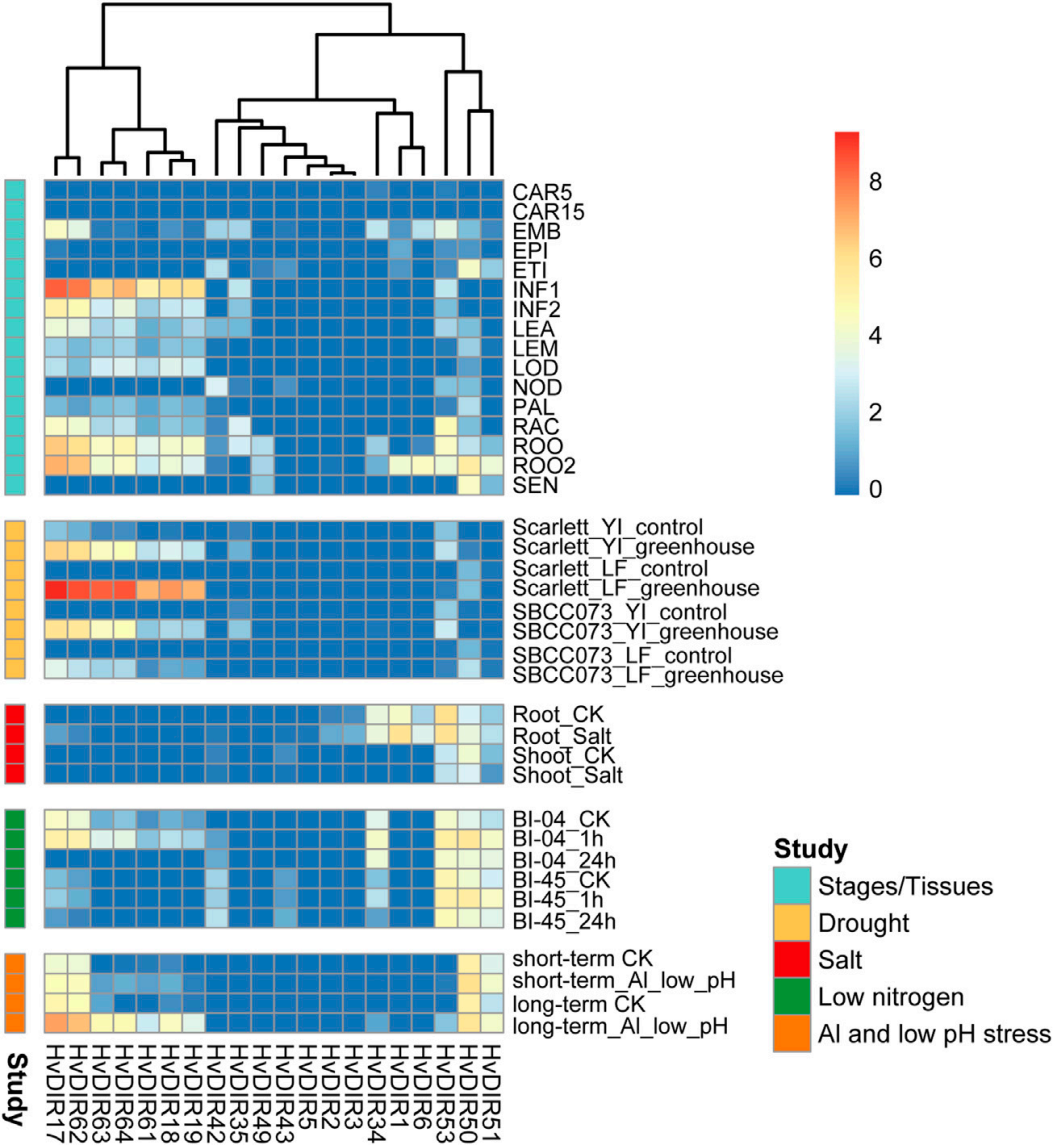
Expression profile of *HvDIR*s at different tissues/stages and in response to various abiotic stresses of barley. FPKM values were normalized by log_2_ (FPKM+1) transform to represent color scores. CAR15/CAR5: Developing grain (15/5 days after pollination); EMB: Embryonic tissue (4 days); EPI: Epidermal strips (4 weeks after pollination); ETI: Etiolated seedling at 10 days old after planting; INF1, Young developing inflorescences with 5 mm; INF2, Developing inflorescences with 1 cm; LEA, 10 cm shoots from seedlings; LEM, Inflorescences, lemma (6 weeks after pollination); LOD, Inflorescences, lodicule (6 weeks after pollination); NOD, Developing tillers at third stem internode (6 weeks after pollination); PAL, Dissected inflorescences, palea (6 weeks after pollination); RAC, Inflorescences, rachis (5 weeks after pollination); ROO, Roots from the seedlings at 17 and 28 days old after planting; ROO2, Roots (4 weeks after pollination); SEN, Senescing leaves (8 weeks after pollination). Yl, Young inflorescence; LF, Leaf.

The biological functions of the expanded *HvDIR*s were investigated by evaluating the tissue-/stage-specific expression patterns in public transcriptome datasets from 16 different tissues/stages (Figure 7). The expression profiles of the expanded genes within or among OGs were somewhat diverse. For example, *HvDIR1* and *HvDIR6* were predominantly expressed in ROO2, but no expression was detected in all tissues/stages for the remaining *DIR*s of OG0001516. Additionally, *HvDIR35* and *HvDIR53* of OG0002727 showed similar expression landscape and were preferentially expressed in RAC and ROO, while the expression of *HvDIR34* was low or absent in most samples. In OG0002286, *HvDIR49*, *HvDIR50*, and *HvDIR51* tended to be highly expressed in ROO, ROO2, and SEN. However, *HvDIR50*, showed high expression level in ETI with an FPKM value of 20.27. Two distinct expression clusters were observed for seven *HvDIR*s within OG0000960, which formed two tandem repeat blocks. Among them, *HvDIR17* and *HvDIR62* showed exceedingly high expression levels in ROO, ROO2, and INF1 (FPKM >65). The remaining five *HvDIR*s (*HvDIR18*, *HvDIR19*, *HvDIR61*, *HvDIR63*, and *HvDIR64*) showed similar tissue-/stage-specific expression profile with relatively lower expression.

The expression patterns in response to drought combined with heat was analyzed in two different barley genotypes, namely a landrace (SBCC073) and a modern cultivar (Scarlett). The *HvDIR*s of OG0000960 were highly induced by stress treatment (Figure 7). For example, *HvDIR17* tended to be silent or lowly expressed under untreated condition, but it was significantly highly expressed in response to drought combined with heat. We further analyzed the expression landscape of *HvDIR*s under salt stress. Compared with the unstressed control, *HvDIR*1 and *HvDIR6* showed a 3.15- and 2.60-fold increase in expression in the root zone. In comparison, *HvDIR50* showed 2.06-fold downregulation. Furthermore, the expanded *HvDIR*s were significant induced at the initial stage (1-h) of low nitrogen, which we termed as short-term response. For instance, the expression of *HvDIR50* (5.26-fold), *HvDIR63* (6.24-fold), and *HvDIR64* (4.34-fold) was highly upregulated at 1-h treatment, but no significant difference in the rate of change could be found at 24-h of treatment. In contrast, the expanded *HvDIR*s had a long-term response to low pH together with aluminum treatment. *HvDIR18*, *HvDIR19*, and *HvDIR63* were upregulated more than 20 fold after 7 days of treatment, whereas after 48-h low pH and 24-h Al treatment, these genes were not significantly induced.

### Analysis of miRNA target sites and co-expression network of the expanded *HvDIR*s

The analysis of the miRNA target sites provided valuable information on the miRNA-mediated posttranscriptional regulatory mechanisms. A total of 16 miRNA-*HvDIR* pairs, including 12 *HvDIRs* and 10 miRNAs, were predicted for the expansion-related *HvDIR* (Supplementary Table S14). Most of the miRNAs (10 out of 16 pairs) regulated the expression of *HvDIR*s through targeting the core region of the DIR domain. In addition, seven of the identified miRNA-*HvDIR* pairs were found to be silenced through translation inhibition, while the remaining nine pairs were silenced by guiding mRNA cleavage. These findings revealed the potential regulatory mechanism involved in the posttranscriptional regulation of *HvDIRs*.

*Cis*-elements play critical roles in the transcriptional regulation of genes throughout the life cycle of plants. In total, 40 types of *cis*-regulatory elements were detected in the promoter region of *HvDIR*s and further classified into three categories. A large number of light-responsive elements were identified, ranking as the most abundant category. We also obtained 13 types of hormone-responsive elements, such gibberellin-responsive elements (TATC-box, GARE-motif, and P-box), auxin-responsive elements (TGA-element, TGA-box, and AuxRR-core) and salicylic acid-responsive elements (TCA-element). In addition, 12 types of abiotic and biotic stress-related *cis*-elements were observed. It should be noted that 40 drought stress-related elements (MBS, myeloblastosis binding site), 38 cold stress-related elements (LTR, low temperature responsive), and 15 wound-responsive elements (WUN-motif) were identified in the promoter region of 12, 6, and 7 expanded *HvDIR*s. These findings suggested that the expanded *HvDIR*s might be involved in hormone signal transduction, growth and development, and various stress adaptation mechanisms in barley (Supplementary Table S15).

**FIGURE 8:**
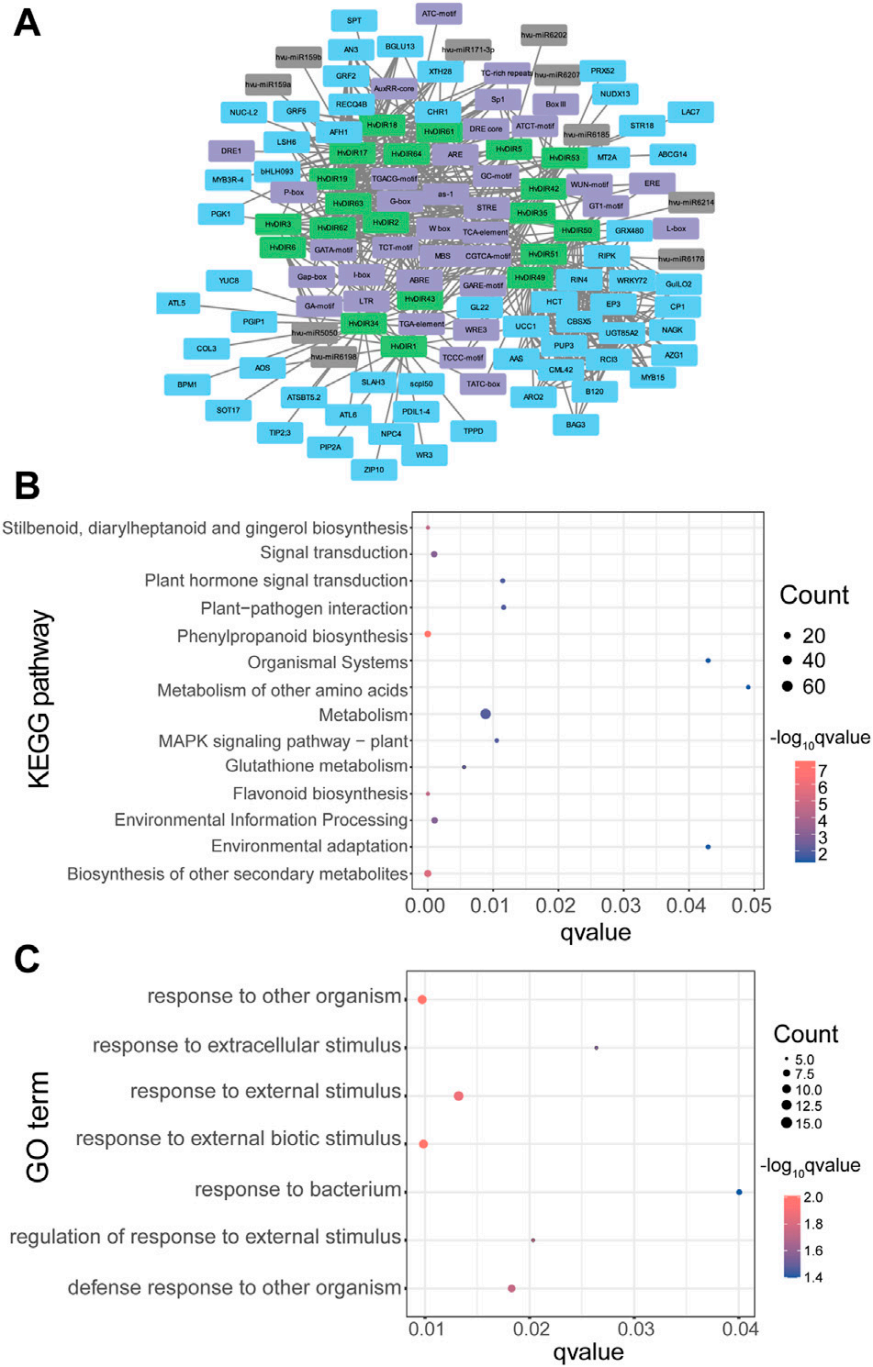
**(A)** The *cis-*elements, miRNA, and co-expression network of *HvDIR*s; **(B)** KEGG pathway enrichment analysis of *HvDIR*s co-expressed genes; **(C)** GO term enrichment analysis of *HvDIR*s co-expressed genes.

Gene co-expression network algorithms have become an effective tool for finding potential regulatory pathways. The WGCNA algorithm found a total of 250 links consisting of 13 expanded *HvDIR*s and 60 co-expressed genes (Figure 8A). Notably, *AN3*, *bHLH093*, *GRF2*, *LSH6*, and *RECQ4B* were all found to be co-expressed with each of 7 *HvDIR*s, suggesting the potential of these genes as central hub genes. The *HvDIR* co-expressed genes were significantly enriched in various KEGG pathways, including phenylpropanoid biosynthesis (KO00940), biosynthesis of other secondary metabolites (KO09110), flavonoid biosynthesis (KO00941), and signal transduction (KO09132) (Figure 8B, Supplementary Table S16). GO term enrichment analysis of the genes co-expressed with *HvDIR*s revealed that they were highly enriched in the terms associated with response processes, such as response to other organism (GO: 0051707), response to external biotic stimulus (GO:0043207), response to extracellular stimulus (GO: 0009991), and response to bacterium (GO: 0009617). (Figure 8C, Supplementary Table S17).

## Discussion

### The *DIR* gene family is expanded in barley

A higher percentage of duplication events has occurred in plant genomes than in other eukaryotes (Lockton and Gaut, 2005). With the availability of multiple genome assemblies, gene family expansion and contraction have been widely found in various plant species, such as the 252 expanded OGs identified in *Olea europaea* cv “*Arbequina*” (Rao et al., 2021), 2,874 in *Senna tora* (Kang et al., 2020), 1,078 in *A. thaliana*, 1,300 in rice, and 497 in *Anthoceros angustus* (Zhang et al., 2020). Due to its large genome size and high transposon content (Schulte et al., 2009), there has been a server lag in the application of these advances in barley research. In recent years, the first released barley reference genome assembly (Mayer et al., 2012), its subsequent improvements (Mascher et al., 2017; Monat et al., 2019), and the most updated version Morex V3 (Mascher et al., 2021), have laid the foundation for a comprehensive understanding of duplication patterns in barley. A total of 289,803 protein-coding genes from eight genomes/subgenomes were assigned into 31,750 OGs using OrthoFinder, an alignment-based algorithm that infers OGs across various species. Among them, 1,113 and 6,739 OGs were found apparently expanded and contracted in barley, respectively. An intriguing feature of the barley genome is the expansion of a series of genes that were functionally associated with plant growth and development processes, and response to hormone, biotic and abiotic stresses. These gene expansions might form the basis of the excellent adaptation of barley to its habitat.

Notably among these expansions is the *DIR* gene family. The DIR proteins are involved in the biosynthesis of lignans, and play a crucial role against abiotic and biotic stresses in plants (Khan et al., 2018). The *DIR* gene family has been well-identified in various land plants (Liao et al., 2017; Khan et al., 2018; Ma et al., 2021) (Supplementary Table S18). In this study, a total of 64 *HvDIR*s, which was far more than that of other plant species, were identified in the barley genome. Based on the classification described in a previous study (Liao et al., 2017), the DIR proteins were divided into five subfamilies. Subfamily DIR-a and DIR-b/d have comparable numbers of DIR proteins from barley, *A. thaliana*, and rice, whereas subfamilies DIR-c and DIR-g do not contain DIR proteins from *A. thaliana*, indicating that these monocot-specific subfamilies contributed greatly to the expansion of DIR proteins in monocot species. In addition, a total of 28 barley DIR proteins were assigned into the DIR-c subfamily, whereas the smaller number, with 13 *OsDIR*s, was identified in rice. Our results demonstrated that the monocot-specific DIR-c subfamily might have undergone continuous expansion after the divergence between monocot and dicot in barley.

### Tandem duplication contributes greatly to the expansion of *HvDIR*s

A gene family consists of a series of similar genes that come from the same ancestor, and gene duplication events, such as SSD and WGD, are the driving force for the rapid evolution and expansion of genes in a particular gene family (Cannon et al., 2004). In the barley genome, segmental, and tandem duplication events are largely responsible for the expansion of gene families. Ten and thirteen *HvmTERF*s have undergone tandem and segmental duplications, respectively, accounting for about 38.33% of the total size of barley *mTERF*s (Li et al., 2021b). Tandem duplication was the main force driving the expansion of *HvNRT2.1* (80.00%) (Guo et al., 2020), whereas segmental duplication contributed greatly for the expansion of *HvbHLH* (42.55%) (Ke et al., 2020). In this study, 17 and 27 *HvDIR*s were found to be involved in segmental and tandem duplications, respectively, suggesting that tandem duplication contributed largely to the expansion of *HvDIR*s. It should be noted that the expansion of OG0000960 (24 segmental duplications and 2 tandem blocks), OG0002727 (4 segmental duplications and 1 tandem block), OG0002286 (0 segmental duplications and 1 tandem block), and OG0017605 (0 segmental duplications and 1 tandem block) were all associated with SSDs. The 7 *HvDIR*s of OG0000960 and 5 *HvDIR*s of OG0001516 were grouped into DIR-c, suggesting that tandem duplication was responsible to the expansion of the DIR-c subfamily. Most (32%) of the duplicated genes shared high sequence identity (>90%), indicating the occurrence of recent duplications. Moreover, the Ka/Ks values of the duplicated gene pairs were lower than one, suggesting that they all underwent purifying selection.

### The expanded *HvDIR*s experienced severe genetic bottleneck during barley domestication

Domestication is the genetic modification of its wild ancestor driven by human needs (Purugganan, 2019). The process of domestication results in several stereotypical changes and is collectively termed as “domestication syndrome” (Parsons et al., 2020). A suite of traits corresponding to “domestication”, such as grain shattering (Pourkheirandish et al., 2015), caryopsis (Taketa et al., 2008), and spike morphology spike (Komatsuda et al., 2007; Bull et al., 2017), have been identified and the related genes have been isolated in barley.

The genetic diversity was continuously narrowed down in most domesticated plants due to the domestication bottleneck (Buckler et al., 2001; Yang et al., 2022). As a consequence of the limited gene pool from its wild ancestor, this severe bottleneck affects throughout the whole genome (Wright et al., 2005). A 27% reduction of nucleotide diversity from wild barley to landraces was found across the barley genome (Russell et al., 2016). However, the genetic diversity varied greatly for certain gene families. For instance, the estimated genetic bottleneck was 22.5% for barley adaptive genes, 18.2% for disease resistance genes (Fu, 2012), and 20% for housekeeping genes (Ding et al., 2020). The nucleotide reduction of the expanded *HvDIR*s was 45.20% (wild barley 0.3281, landraces: 0.1798), indicating a severe genetic bottleneck that reduced the nucleotide diversity of alleles for the expanded *HvDIR*s. Consistent with the nucleotide diversity analysis, the haplotype composition in wild barley was richer than that in landraces, and haplotype divergence has occurred passing from wild to cultivated populations. These results indicated that the *HvDIR*s experienced a severe bottleneck during the domestication of barley, and might be domestication-related genes.

### The expanded *HvDIR*s might have multiple biological functions, especially in response to environmental stimuli

Following gene duplication and loss, environmental adaptation and plant speciation appeared to have occurred through a combination of both regulatory and structural alterations in one or more duplicated genes (Hoekstra and Coyne, 2007). Recent developments in sequencing technologies have led researchers to make great progress in understanding the underlying gene evolution process, which has revealed critical insights into the relationships between the expansion of gene families and sub-/neo-functionalization (Harris and Hofmann, 2015). An extended gene family in Mango is the chalcone synthase (*CHS*) family, and certain genes within this family are likely to be involved in the biosynthesis of urushiols and related phenols (Wang et al., 2020). The leaf and stem vascular regulator *BRI1-BRL* gene family was found to be expanded in *Adiantum capillus-veneris* L. and functionally conserved for vascular development regulation in euphyllophytes, suggesting that complicated vascular system formation led to the origin of true leaves or euphylls (Fang et al., 2022).

Spatiotemporal regulation of gene expression could be controlled by *cis*-acting regulatory elements within the promoter region of genes. Many *cis*-elements within the promoter regions of *HvDIR*s were identified, which were further categorized into three groups, namely hormone induction, light, and stress response. These results indicated that the expanded *HvDIR*s might participate in various biological processes.

The expression patterns of *HvDIR*s in specific tissues or at specific stages lay the foundation for their possible functions. For example, *HvDIR1* and *HvDIR6* showed preferential expression in ROO2; *HvDIR35* and *HvDIR53* were predominant in RAC and ROO. Moreover, *HvDIR50* was highly expressed in ETI, suggesting that these *HvDIR*s might be involved in plant organogenesis in barley. Due to their sessile nature, terrestrial plants are constantly exposed to a multitude environmental perturbation, such as extreme temperatures, high salinity, drought, and pathogen infection (Zhu, 2016; Saijo and Loo, 2020). To optimize growth under such environmental challenges, plants have evolved sophisticated mechanism to perceive internal and external signals and coordinate plant growth and development (Verma et al., 2016). DIR proteins were involved in lignan biosynthesis, and play essential roles against abiotic and biotic stresses in plants (Khan et al., 2018). Our study analyzed the expression patterns of the expanded *HvDIRs* in response to drought, salt, low nitrogen, and low PH (Figure 8). *HvDIR*s from OG0000960 were found to be extremely highly expressed in response to drought stress. A suite of MBS *cis*-regulatory elements related to drought inducibility were predicted within their promoter regions, indicating their potential roles in response to drought stress. *HvDIR1* and *HvDIR6* from OG0001516 were highly induced under salt treatment. Furthermore, the expanded *HvDIR*s tended to perform short-term functions under low nitrogen, but long-term functions in response to low pH and Al stresses. Notably, *HvDIR18*, *HvDIR19*, and *HvDIR63* were found to be significantly induced by various stresses, including drought, low nitrogen, as well as low pH with Al, which could serve as excellent targets for genetic isolation and functional characterization of barley.

The WGCNA is a system biology method that is widely applied to identify highly correlated modules and examine the co-expression patterns among genes (Xu et al., 2018). Lignification is associated with the structural resistance to pathogens and it plays essential roles against pathogens attack by inhibiting microbe-derived degradative enzymes (Lee et al., 2019; Yadav et al., 2020). The involvement of *DIRs* in the biosynthesis of lignans suggested their potential roles in plant–pathogen interaction. Despite the lack of direct validation of transcriptome data of biotic stresses, the co-expression network we constructed can also serve as reference for the function of *HvDIR*s. KEGG pathway and GO enrichment analysis indicated that the expanded *HvDIR*s of the network correlated with gene expression and are involved in response to multiple biotic and abiotic stresses. We ultimately constructed a comprehensive regulatory network of the expanded *HvDIR*s by integrating miRNA, *cis*-element, and co-expressed genes, which provides clues to identify the gene regulatory mechanisms underlying their biological functions.

## Data Availability

The original contributions presented in the study are included in the article/Supplementary Material, further inquiries can be directed to the corresponding author.

## Supplementary material

SUPPLEMENTARY FIGURE S1 KEGG and GO analyses of expansion genes in barley. GO term enrichment analysis (The first 20 biological process with corrected p-value <0.05) of expansion genes in barley; **(B)** KEGG pathway analysis analysis (The first 20 biological process with corrected p-value <0.05) of expansion genes in barley.

SUPPLEMENTARY FIGURE S2 Distribution of HvDIR genes on barley chromosomes. Chromosome number is shown at the top of each chromosome. The scale (Mb) is indicated on the left.

SUPPLEMENTARY FIGURE S3 Location of 17 important cis-acting elements in the 1.5 KB upstream HvDIR coding region and represented by different colors.

## Glossary

**ABA**: abscisic acid
**BLAST**: basic local alignment search tool
**CHS**: chalcone synthase
**CDS**: Coding Sequence
**DIR**: dirigent
**ESB1**: enhanced suberin1
**FPKM**: fragments per kilobase of transcript per million fragments mapped reads
**GRAVY**: grand average of hydropathicity
**GSDS**: gene structure display server
**GO**: gene ontology
**GTF**: gene transfer format
**HMM**: The hidden Markov model
**Ka**: nonsynonymous
**Ks**: synonymous
**KEGG**: kyoto encyclopedia of genes and genomes
**LTR**: low temperature responsive
**MYA**: million years ago
**MG2C**: MapGene2Chrom
**MW**: molecular weight
**MAF**: minor allele frequency
**MBS**: myeloblastosis binding site
**NCBI**: National Center for Biotechnology Information
**NCBI-CDD**: National Center for Biotechnology Information Conserved Domain Database
**NJ**: neighbor joining
**OG**: orthologous group
**PCA**: principal component analysis
**pI**: theoretical isoelectric point
**RNA-seq**: RNA sequencing
**SNP**: single nucleotide polymorphism
**SMART**: simple modular architecture research tool
**SRA**: sequence read archive
**SSD**: small-scale duplication
WGD: whole genome duplication
WGCNA: weighted gene co-expression network analysis
