# Supplementary figures and images for: The barley DIR gene family: An expanded gene family that is involved in stress responses
Source: Front Genet. 2022 Nov 2;13:1042772. doi: 10.3389/fgene.2022.1042772 (PMC9667096; doi:10.3389/fgene.2022.1042772)

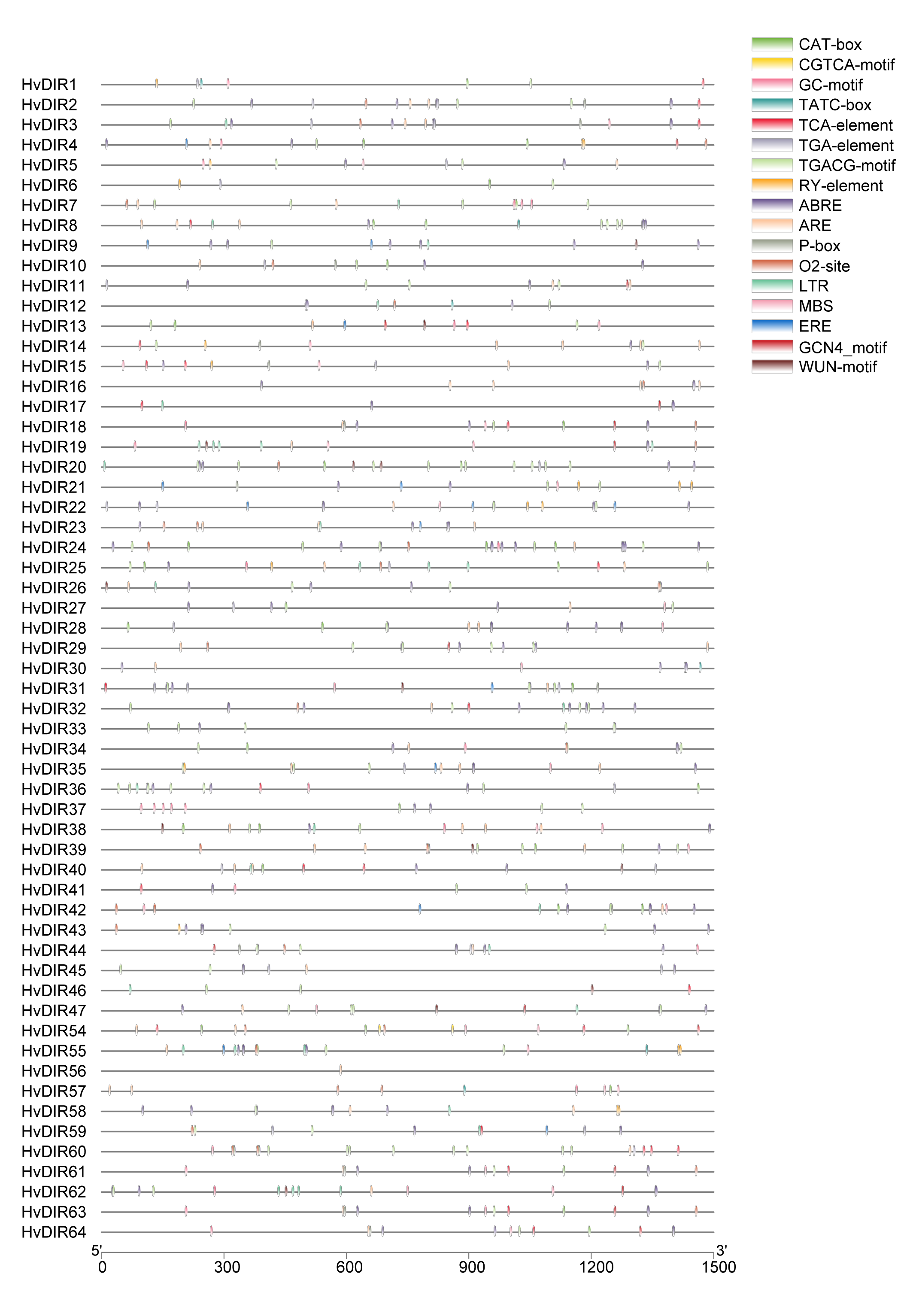

Supplement: Supplementary file 1 [file Image3.TIF]

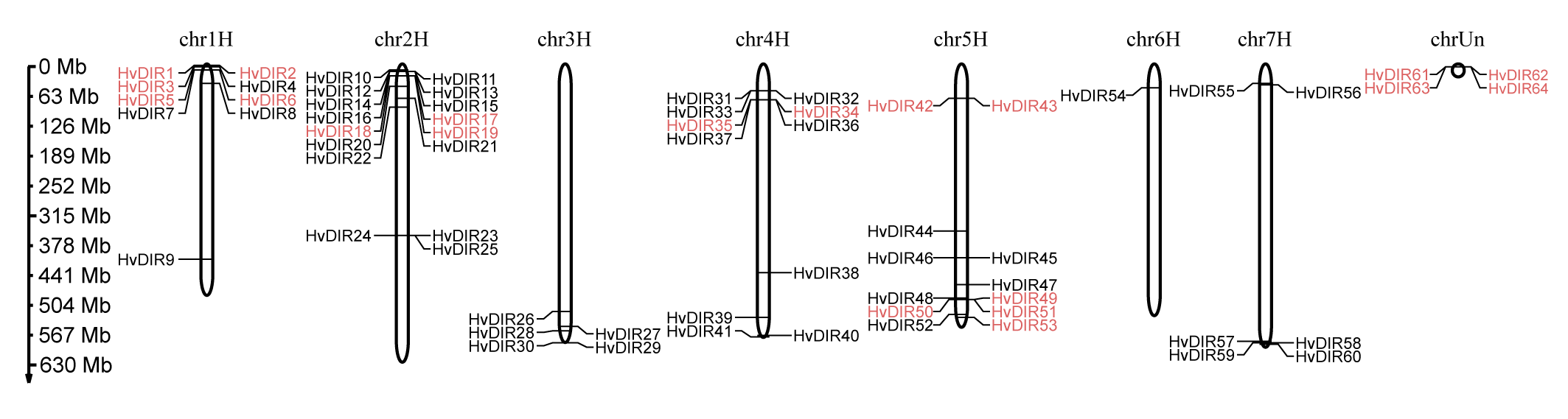

Supplement: Supplementary file 2 [file Image2.TIF]

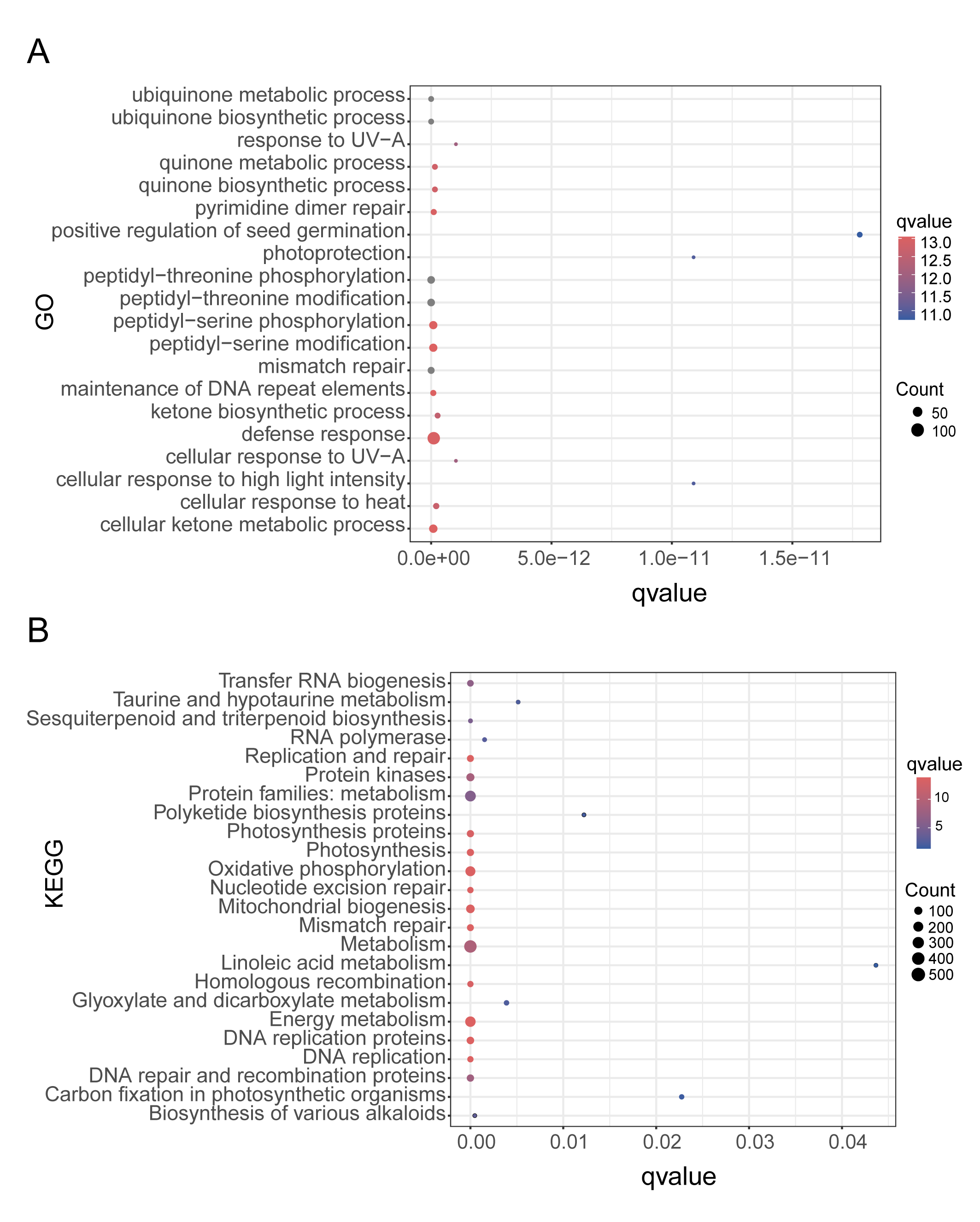

Supplement: Supplementary file 3 [file Image1.TIF]
